# Opportunities and challenges in sharing and reusing genomic interval data

**DOI:** 10.3389/fgene.2023.1155809

**Published:** 2023-03-20

**Authors:** Bingjie Xue, Oleksandr Khoroshevskyi, R. Ariel Gomez, Nathan C. Sheffield

**Affiliations:** ^1^ Center for Public Health Genomics, School of Medicine, University of Virginia, Charlottesville, VA, United States; ^2^ Department of Biomedical Engineering, School of Medicine, University of Virginia, Charlottesville, VA, United States; ^3^ Child Health Research Center, School of Medicine, University of Virginia, Charlottesville, VA, United States; ^4^ School of Data Science, University of Virginia, Charlottesville, VA, United States; ^5^ Department of Public Health Sciences, School of Medicine, University of Virginia, Charlottesville, VA, United States; ^6^ Department of Biochemistry and Molecular Genetics, School of Medicine, University of Virginia, Charlottesville, VA, United States

**Keywords:** data sharing and reuse, genomic intervals, genomic regions, epigenome analysis, data integration

## 1 Introduction

**FIGURE 1:**
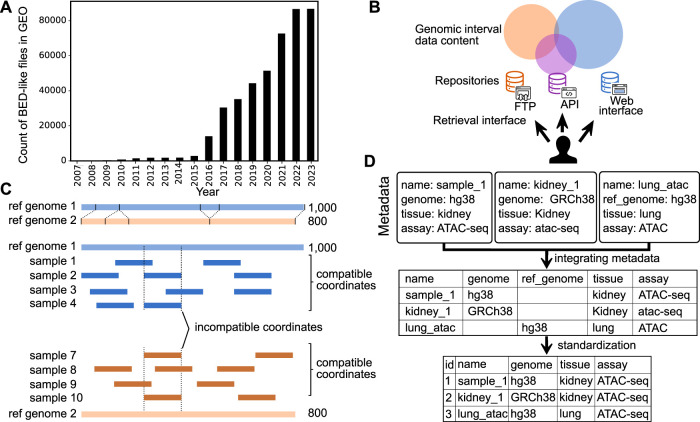
Overview of challenges in sharing and reusing genomic interval data. **(A)** Growth in number of BED-like files in GEO (files on either GEO series or sample entries with names including bed, bigBed, narrowPeak, or broadPeak, and size less than 25 mb). **(B)** Illustration of identifying and retrieving genomic interval data among fragmented repositories with different data retrieval mechanisms. **(C)** Demonstration of the challenge to integrating genomic interval data across different reference genomes. **(D)** Integration of metadata with and without standardization.

Major advances in genome sequencing technology have driven a dramatic increase in the production of epigenome data (ENCODE Consortium, 2012; Sheffield and Furey, 2012; Martens and Stunnenberg, 2013; Kundaje et al., 2015; Sheffield and Bock, 2016; Zheng et al., 2018). Epigenomic data result from various experiments, such as chromatin immunoprecipitation, DNA methylation, and chromatin accessibility assays. The resulting data is often represented as genome signals, or “wiggle tracks,” which are summarized into regions, or genomic intervals, stored in BED (browser extensible data) file format (Kent et al., 2002). Genomic interval data is useful for a variety of biological questions, such as identifying genetic variants associated with diseases, determining the function of genes and pathways, understanding gene-by-environment effects, studying the underlying mechanisms of disease, and developing personalized treatments. To leverage the value of this public genomic interval data, we must share it broadly, and the number of publicly available genomic interval files has risen quickly; more than 80,000 BED files are now available from the Gene Expression Omnibus (GEO) (Figure 1A). However, despite recent progress in scale and access, reusing genomic interval data is still challenging.

Reusing large-scale data faces many general challenges. Here, we focus specifically on integrating genomic interval data from different sources to enable researchers to compare and apply downstream analysis on those data. We outline five challenges with reusing genomic interval data: 1) identifying and retrieving relevant data; 2) identifying and integrating across reference genomes; 3) standardizing BED format; 4) integrating mixed-quality and mixed-process data; and 5) standardizing metadata. We argue that addressing these challenges will reduce barriers to sharing genomic interval data and lead to increased biomedical discovery.

## 2 Challenges in reusing genomic intervals

### 2.1 Identifying and retrieving relevant data

The first challenge in reusing genomic interval data is identifying and retrieving relevant data. Despite the scale and importance of existing genomic interval resources, it is difficult to identify and retrieve relevant data for the following three reasons: 1) data repositories are fragmented; 2) search methods rely on author-provided metadata; and 3) genomic interval repositories lack a standard API for retrieving data (Figure 1B). First, existing interval databases are fragmented and restricted to subsets of the available genomic interval data. For example, some databases are restricted by ethnicity, by research project, or only contain certain data types. This can make it difficult to find or retrieve data of interest by multiplying the places to search. One solution is to build integrative databases; for example, the International Human Epigenome Consortium (IHEC) has made efforts to integrate and share data from major national endeavors such as United States ENCODE and NIH Roadmap, European Blueprint, Canadian CEEHRC, German DEEP, Korean KNIH, and China’s EpiHK (Stunnenberg et al., 2016). Other examples include the Cistrome Data Browser (Cistrome DB) (Zheng et al., 2018) and ChIP-Atlas (Zou et al., 2022), which integrate interval data across ChIP-seq, ATAC-seq, DNase-seq, and Bisulfite-Seq data. These projects reduce fragmentation, which facilitates reuse; however, integrating is incomplete—the community still lacks a comprehensive source of all genomic interval data.

The second problem that makes identifying and retrieving relevant data a challenge is that existing search methods mostly rely on metadata matching only, which may result in an incomplete or incorrect list of relevant results. For example, the Gene Expression Omnibus (GEO) (Barrett et al., 2013) is probably the largest repository of genomic interval data, containing tens of thousands of biological samples. The data search within GEO is limited to keywords within the metadata provided by the study’s authors. This can be problematic if the metadata is missing or incorrect, or simply uses different synonyms. A variety of attempts to restructure the GEO metadata have tried to improve the situation (Davis and Meltzer, 2007; Choudhary, 2019; Khoroshevskyi et al., 2023). One promising approach is to use machine learning to identify patterns and features and allow data with missing or incorrect metadata to be retained in search results if they have similar characteristics to data containing the relevant keywords (Leipzig et al., 2021; Garcia et al., 2022). New approaches that build on natural language processing to associate genomic regions with human-friendly keywords is an opportunity for future development.

Finally, retrieving relevant genomic interval data is challenging due to the lack of a simple, universal programmatic data retrieval method. Genomic interval data sources generally provide user-friendly web interfaces, which are valuable for browsing, but manually searching and downloading data can be tedious. To address this issue, tools like DeepBlue and FILER provide easy programmatic data retrieval *via* application programming interfaces (API) (Albrecht et al., 2016; Kuksa et al., 2022). However, these databases only contain a subset of the available genomic interval data, and there is a need for a unified platform and standardized, cross-platform APIs that allow easy access to all existing resources.

### 2.2 Identifying and integrating across reference genomes

The second challenge is identifying the reference genome. Genomic interval annotations are only comparable if they are defined on the same coordinate system (Figure 1C). Yet, despite this importance, it remains difficult to identify and integrate reference genomes across genomic interval data because 1) genome assembly identifiers are ambiguous; and 2) reference genome information is not contained in the genomic interval data file itself.

First, the ambiguity in the genome assembly identifiers can make identifying the reference genome of genomic interval data difficult. For example, the same human-readable identifier may refer to many variations of the human genome. A solution to this problem is to use unique identifiers that unambiguously identify a particular assembly (Kitts et al., 2015). However, this approach relies on a central authority, and different consortia may use different genome identifier systems, leading to inconsistencies and potential errors in the analysis. To avoid errors in identifying and comparing reference genomes for genomic interval data, refgenie developed a new system to establish the identity of a genome based on the refget protocol and using the digest of reference genome content as the genome identifier (Stolarczyk et al., 2021; Yates et al., 2021). This approach allows users to confirm *via* computation the identity of the reference genome used to generate the genomic interval data across systems. However, this approach is not yet widespread, and many reference genome identifiers remain ambiguous.

Another aspect of this challenge is that reference genome information is not contained within the genomic interval data files themselves. The reference genome a BED file annotates is usually given in its metadata or as part of the file name. Unfortunately, these sources of information may become disconnected, and also make it difficult to accurately integrate and annotate genomic interval data without additional metadata. Therefore, efforts are needed to develop a standard that encodes the reference genome identity in the genomic interval data file.

### 2.3 Standardizing BED format

A third challenge is inconsistent file formats. Genomic interval data is typically summarized in BED format, which, according to the standard, contains three required fields (seqname, start, and end) and nine optional fields (Kent et al., 2002). BED-like formats, such as the narrowPeak and broadPeak formats, are even more flexible and can have different information. In addition, BED files are frequently adapted, and dozens of possibly undocumented variations exist. For example, the signal values that are typically represented in the WIG file can be a custom field that may not be encoded similarly across BED files in an integrative study. In addition, there is also GFF format, which also encodes genomic locations, but differs in column order. This inconsistency makes integrating BED files challenging for two reasons: First, some BED files may contain optional or custom fields that are absent in other BED files. Second, different BED files may use different names for the custom fields of the same information. The different file formats can make integrating and comparing data from different sources difficult, hindering the reuse of genomic interval data.

### 2.4 Integrating mixed-quality and mixed-process data

A fourth challenge is integrating data with different quality, completeness, or processing steps. The quality of the data and its computational processing is frequently unknown, because sample quality may not be included in interval files and pipeline parameters used to process raw sequencing data are often unknown. Data with different sources or analysis steps is challenging to integrate, which hinders building upon previous work. To ensure the quality and uniformity of the data, one solution is to apply either standardized quality control (QC) or entire data preprocessing pipelines, or both. For example, the Cistrome DB applied the ChiLin pipeline for chromatin profiling data analysis and quality control (QC) using a set of QC criteria, including uniquely mapped reads, PCR bottleneck coefficient, and the FRiP score (Qin et al., 2016; Zheng et al., 2018). This approach creates universal QC scores and standardizes the pipeline, but it requires far greater resources than simply re-using existing published genomic interval sets. Furthermore, there are many different steps in a raw sequencing processing, such as alignment, peak calling, and signal track generation. This restricts the standardized pipeline approach to allowable data types.

In addition to the mixed-quality data, we also want to filter out the duplicated data when reusing genomic interval data. Because there are no general-purpose global identifiers for BED files, an analysis that scrapes BED data from multiple sources is likely to collect duplicated data. Using a checksum-based approach could help to identify files that are identical, but differences in whitespace, columns, or other manipulations can fool this method. A more effective approach could assign identifiability based on the actual region coordinates, rather than file checksums alone.

### 2.5 Standardizing metadata

The last challenge is integrating metadata across sources. Currently available genomic interval metadata faces three rampant problems: 1) non-overlapping attribute names, 2) incomplete data, and 3) lack of controlled vocabulary (Figure 1D). These classic challenges apply to all types of biomedical metadata but are particularly pronounced for genomic intervals due to the diversity of data sources and processing. First, different sources of genomic interval data may use different names for the same attribute, making it difficult for researchers to integrate the data. For example, parsing the metadata from GEO results in a sporadic table with multiple columns of the same attribute because the authors of the different studies use different names for the same attribute. Second, some sources may provide only some of the metadata required for integration, making it difficult to fully understand the context and relevance of the data. In the context of genomic interval data, metadata should include information such as experimental assay (e.g., ChIP-seq or ATAC-seq), type of genomic records (e.g., narrow peaks, broad peaks, and gene models), biological sources, and the reference genome. Third, integrating genomic interval data can be particularly challenging when the metadata values use different terminology. Using a controlled vocabulary helps ensure that metadata is consistently and accurately described, making it easier to search for, retrieve, and analyze the data. Controlled vocabularies can also help reduce confusion and ambiguity, as they provide a clear, standardized set of terms that can describe the data. However, their use is sporadic at best. These three issues can make it challenging to effectively reuse and integrate genomic interval data from multiple sources, limiting the potential benefits of reusing such data. One approach to address this challenge is to reprocess published data to produce curated databases with uniform processing and standardized metadata annotation (Albrecht et al., 2016; Kuksa et al., 2022).

## 3 Discussion

There are many challenges to handling growing data resources across disciplines. In this paper, we identified challenges specific to genomic interval data. Genomic interval data are a major resource for biological research, but the above challenges with sharing and reusing genomic interval data prevent the community from making the most of it. With the overwhelming scale of the existing genomic interval data, we need platforms and databases that can efficiently manage these resources. To overcome the challenges, efforts including integrating genomic interval data from different data sources, developing new methods to identify reference genomes, providing standardized data processing and QC pipelines, standardizing metadata, and designing easy-to-use APIs for data access. In the future, we must continue to invest in this area to develop tools that aggregate existing large scale genomic interval data, improve data standardization and browsing, and enhance discoverability and programmatic retrievability. This will allow us to fully leverage the value of genomic interval data and improve research efficiency, effectiveness, reproducibility, and credibility.
